# Evaluation of a Fully Automatic Deep Learning-Based Method for the Measurement of Psoas Muscle Area

**DOI:** 10.3389/fnut.2022.781860

**Published:** 2022-05-12

**Authors:** Dennis Van Erck, Pim Moeskops, Josje D. Schoufour, Peter J. M. Weijs, Wilma J. M. Scholte Op Reimer, Martijn S. Van Mourik, Yvonne C. Janmaat, R. Nils Planken, Marije Vis, Jan Baan, Robert Hemke, Ivana Išgum, José P. Henriques, Bob D. De Vos, Ronak Delewi

**Affiliations:** ^1^Department of Cardiology, Amsterdam UMC, University of Amsterdam, Amsterdam, Netherlands; ^2^Faculty of Sports and Nutrition, Center of Expertise Urban Vitality, Amsterdam University of Applied Sciences, Amsterdam, Netherlands; ^3^Quantib-U, Rotterdam, Netherlands; ^4^Faculty of Health, Center of Expertise Urban Vitality, Amsterdam University of Applied Science, Amsterdam, Netherlands; ^5^HU University of Applied Sciences, Research Group Chronic Diseases, Utrecht, Netherlands; ^6^Department of Radiology and Nuclear Medicine, Amsterdam UMC, University of Amsterdam, Amsterdam, Netherslands; ^7^Department of Biomedical Engineering and Physics, Amsterdam UMC, University of Amsterdam, Amsterdam, Netherlands

**Keywords:** computed tomography, muscle assessment, artificial intelligence, psoas muscle area (PMA), body composition, sarcopenia

## Abstract

**Background:**

Manual muscle mass assessment based on Computed Tomography (CT) scans is recognized as a good marker for malnutrition, sarcopenia, and adverse outcomes. However, manual muscle mass analysis is cumbersome and time consuming. An accurate fully automated method is needed. In this study, we evaluate if manual psoas annotation can be substituted by a fully automatic deep learning-based method.

**Methods:**

This study included a cohort of 583 patients with severe aortic valve stenosis planned to undergo Transcatheter Aortic Valve Replacement (TAVR). Psoas muscle area was annotated manually on the CT scan at the height of lumbar vertebra 3 (L3). The deep learning-based method mimics this approach by first determining the L3 level and subsequently segmenting the psoas at that level. The fully automatic approach was evaluated as well as segmentation and slice selection, using average bias 95% limits of agreement, Intraclass Correlation Coefficient (ICC) and within-subject Coefficient of Variation (CV). To evaluate performance of the slice selection visual inspection was performed. To evaluate segmentation Dice index was computed between the manual and automatic segmentations (0 = no overlap, 1 = perfect overlap).

**Results:**

Included patients had a mean age of 81 **±** 6 and 45% was female. The fully automatic method showed a bias and limits of agreement of −0.69 [−6.60 to 5.23] cm^2^, an ICC of 0.78 [95% CI: 0.74–0.82] and a within-subject CV of 11.2% [95% CI: 10.2–12.2]. For slice selection, 84% of the selections were on the same vertebra between methods, bias and limits of agreement was 3.4 [−24.5 to 31.4] mm. The Dice index for segmentation was 0.93 **±** 0.04, bias and limits of agreement was −0.55 [1.71–2.80] cm^2^.

**Conclusion:**

Fully automatic assessment of psoas muscle area demonstrates accurate performance at the L3 level in CT images. It is a reliable tool that offers great opportunities for analysis in large scale studies and in clinical applications.

## Introduction

Low muscle mass is a key criteria for diagnoses of malnutrition and sarcopenia (1, 2). It furthermore is a predictor for functional decline, falls, rehospitalization and higher mortality rates at mid-term to long-term follow-up (3). When low muscle mass is diagnosed timely, nutrition and exercise interventions can be implemented to mitigate muscle loss and subsequently improve clinical prognosis (4, 5). The gold standard for determination of muscle mass is the measurement of muscle area on the Computed Tomography (CT) scan (6). CT scans are frequently available in daily clinical practice, but muscle mass measurement for clinical assessment is currently not performed. A limiting factor is the processing method, which requires both expertise and time (7). Therefore, for clinical implementation and large-scale clinical outcome studies a fully automatic method is an unmet need.

Typically, muscle mass is assessed with proxy measures such as total muscle area or psoas muscle area determined in a single axial slice at level of lumbar vertebra 3 (L3) (6, 8). Both are highly correlated with total body muscle mass (9). Furthermore, the two areas show a good predictive ability for various clinical outcomes including major surgical complications, quality of life and mortality (6). It takes two steps to calculate total muscle area or psoas muscle area on an axial slice. First, the correct slice has to be selected and second, the muscle area of interest has to be identified, a process which is known as segmentation (10). Currently both processes are performed manually by trained researchers requiring approximately 5 min for slice selection and 10 min for segmentation per scan (6, 11).

Due to continuous improvements in medical image analysis, deep learning-based methods are able to replace the cumbersome manual process of slice selection and segmentation (12). Several studies have shown promising results for the automatic segmentation of muscle area on a single axial slice of the CT scan (12–15). However, validated fully automatic methods performing slice selection as well as segmentation is still lacking. The main purpose of this study is to evaluate slice selection and segmentation of the psoas muscle area on the CT scan of a fully automatic deep learning method compared to a manual method.

## Materials and Methods

### Study-Cohort

For this study, data of all patients with severe aortic stenosis that were planned to undergo Transcatheter Aortic Valve Replacement (TAVR) from January 2010 to January 2016 were used (*n* = 651) (16). All patients provided informed consent for the procedure. The institutional review board approved this study with a waiver. The protocol was in accordance with the declaration of Helsinki.

### Data Collection

All patients underwent a CT scan prior to TAVR, with a 64-slice multi-detector scanner (Brilliance, Philips Medical Systems, Cleveland, OH) or a dual source 2 × 192 slice multi-detector scanner (Somatom Force, Siemens, Erlangen, Germany) after intravenous contrast administration. A body scan from cranium to thighs was acquired and reconstructed with at a slice thickness of 1.5 or 3.0 mm. A more detailed description of the method is provided elsewhere (16). Patient characteristics were collected from the prospective Institutional TAVR registry.

### Manual Psoas Measurement

Manual assessment of the psoas muscle area had been previously performed by Van Mourik et al. (16). In short, slices were selected manually in the middle of L3, at the rear end of the vertebral body, using a multiplanar view on the Sante DICOM viewer (version 5.0.4, Santesoft, Athens, Greece). For segmentation Slice-O-Matic software (version 5.0, TomoVision, Montreal, Quebec, Canada) was used. Psoas muscle area was manually segmented with a mouse-operated paint brush selecting pixels with HU values between −29 and 150. Complete segmentation was performed by one researcher (YJ), trained in segmentation of psoas muscle area, blinded for patient characteristics.

### Automatic Psoas Measurement

The automatic assessment was performed by the deep learning-based software of Quantib Body Composition (version 0.1.0, Quantib, Rotterdam, Netherlands) (17). The Quantib Body Composition software is available online for testing^1^ and provides quantification of three muscle areas: psoas muscle, abdominal muscle, long spine muscle, and two fat tissues: visceral fat and subcutaneous fat. Furthermore, features can be calculated from these segmentations such as skeletal muscle density (SMD) or intramuscular adipose tissue (IMAT). The algorithm allows post-hoc decisions for selection thresholds of Hounsfield Units (HU). The narrow field of view in several CT images only allowed accurate measurements of the psoas muscle in this cohort.

The software first resampled CT scans to 5 mm slice thickness. Based on these resampled slices, the L3 slice was automatically selected using a convolutional neural network. Subsequently, segmentation of the muscle and fat area was performed in the selected slice using a second convolutional neural network. To limit the influence of the automatic selection, results were averaged over 5 slices, i.e., 25 mm. Boundaries in Hounsfield Units (HU) between 29 and 150 HU were used for selection of muscle tissue. As output the program provided area of the muscle area and fat area in squared centimeters.

All automatically selected slices as well as all automatically segmented muscles were visually inspected. Three random examples and the three biggest outliers, based on the within-subject Coefficient of Variation (CV) for segmentation and distance from reference for slice selection, are shown and discussed in the result section. The examples and outliers are used as case examples to give an overview of the performance of the Quantib software compared to manual slice selection and segmentation.

### Statistical Analysis

Data of continuous variables were presented as mean ± standard deviation or median and Inter Quartile Range (IQR) depending on distribution. Categorical variables were reported as a frequency and percentage.

The area of the psoas muscle attained with automatic method was compared with the manual method using the mean or median difference, the average bias and 95% limits of agreement, the two-way mixed ICC, the within-subject CV (18), and Bland-Altman analysis (19). Additionally, to get insight into the performance of the method’s two components, the slice selection and segmentation were evaluated separately. Detailed comparison between automatic and manual slice selection was performed with visual inspection by one author (DvE) to determine the number of scans that were not on the same vertebra. Comparison of segmentation was performed using the Dice index, a metric that describes overlap between the two segmentations (0 = no overlap, 1 = perfect overlap). For evaluation of the segmentation the manually selected slices were used.

To investigate the method’s performance, the evaluation is furthermore shown between sexes and for different Body Mass Index (BMI) categories (normal/underweight: BMI = 25, overweight BMI > 25 and = 30 or obese BMI > 30) (13). Differences between groups were calculated with student *t*-test or one-way ANOVA and Tukey’s tests. Significance level was set at *p* < 0.05 and all analysis were performed in R statistical software version 3.6.0.

## Results

### Study Cohort

Of the 651 patients undergoing TAVR, 68 patients were excluded, 36 patients had no CT in their medical records, in 15 patients the lumbar section was not available on the scan, 12 patients had no full body scan at 1.5 or 3 mm and 5 patients had an unusable scan at L3 due to noise or artifacts. The remaining 583 patients had a mean age of 81 ± 6 year and 45% was female. A comprehensive list of patient characteristics is shown in Table 1.

**TABLE 1:** Patient characteristics.

	Total (*n* = 583)	Male (*n* = 264)	Female (*n* = 319)
Age, years, mean ± SD	81 ± 7	81 ± 7	81 ± 8
Height, cm, mean ± SD (*n* = 582)	167 ± 9	174 ± 7	161 ± 7
Weight, kg, mean ± SD	77 ± 15	81 ± 14	73 ± 15
BMI, kg/m^2^, mean ± SD Underweight BMI < 18.5, *n* (%) Normal BMI > 18.5 and < 25, *n* (%) Overweight BMI > 25 and < 30, *n*% Obese BMI > 30, *n*%	27.5 ± 4.9 5 (1) 186 (32) 247 (42) 145 (25)	26.8 ± 4.0 2 (1) 91 (34) 123 (47) 48 (14)	28.1 ± 5.5 3 (1) 95 (30) 124 (39) 97 (30)
BSA, m^2^, mean ± SD (*n* = 582)	1.9 ± 0.2	2.0 ± 0.2	1.8 ± 0.2
NYHA, III/IV, *n* (%)	411 (70)	185 (70)	226 (71)
Diabetes mellitus, *n* (%) (*n* = 582)	175 (30)	87 (33)	88 (28)
PAD, yes, *n* (%)	157 (27)	94 (36)	63 (20)
COPD, yes, *n* (%)	190 (33)	97 (37)	93 (29)
Albumin, g/L, mean ± SD (*n* = 480)	42 ± 4	42 ± 4	42 ± 4
Hemoglobin, mmol/L, Mean ± SD (*n* = 580)	7.9 ± 1.0	8.0 ± 1.1	7.7 ± 0.9
eGFR, mL/min/1.73 m2, mean ± SD (*n* = 578)	66 ± 23	65 ± 23	66 ± 23
STS-riskscore,%, mean ± SD (*n* = 582)	5.3 ± 3.3	5.2 ± 3.4	5.4 ± 3.2
Euroscore II (%), mean ± SD	5.5 ± 4.2	6.3 ± 4.9	4.9 ± 3.4
Left ventricular ejection fraction < 45%, *n* (%), (*n* = 582)	108 (19)	69 (26)	39 (12)
Aortic valve area (cm2), mean ± SD (*n* = 540)	0.82 ± 0.29	0.84 ± 0.20	0.81 ± 0.34
Aortic valve peak gradient (mmHg), mean ± SD (*n* = 562)	68 ± 23	68 ± 22	69 ± 24

*SD, standard deviation; BMI, body mass index; BSA, body surface area; NYHA, New York Heart Association; STS, society of thoracic surgeons; PAD, peripheral arterial disease; eGFR, estimated glomerular filtration rate; COPD, chronic obstructive pulmonary disease.*

### Evaluation of Psoas Measurement

**FIGURE 1:**
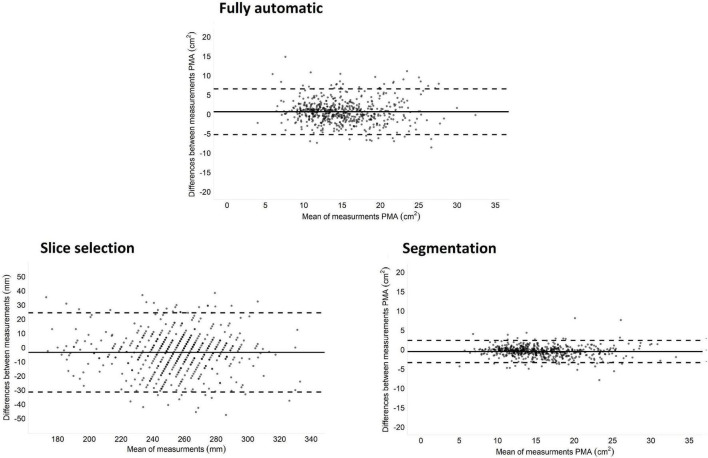
Bland-Altman plots of difference between the manual and automatic method. Fully automatic is automatic slice selection followed by automatic segmentation compared to complete manual slice selection and segmentation. Slice selection is automatic versus manual slice selection. Segmentation is automatic versus manual segmentation on the manual selected slice.

In the fully automatic method, i.e., automatic slice selection followed by automatic segmentation, the average measured psoas muscle area was 15.4 ± 4.6 cm^2^ for the manual method and 14.7 ± 4.7 cm^2^ for the automatic method. Median difference was 1.54 cm^2^ [IQR: 0.78–2.96], and Bland-Altman analysis (Figure 1) showed a bias of −0.69 cm^2^ with a 95% limit of agreement of −6.60 to –5.23 cm^2^. The fully automatic muscle assessment had an ICC of 0.78 [95% CI: 0.74–0.82] and a within-subject CV of 11.2% [95% CI: 10.2–12.2]. Male patients had a significantly lower within-subject CV than female patients, 10.0% [8.6–11.4] vs. 12.2% [10.8–13.6] (*p* = 0.03). No significant differences were seen among the BMI groups.

### Evaluation of L3 Slice Selection

**FIGURE 2:**
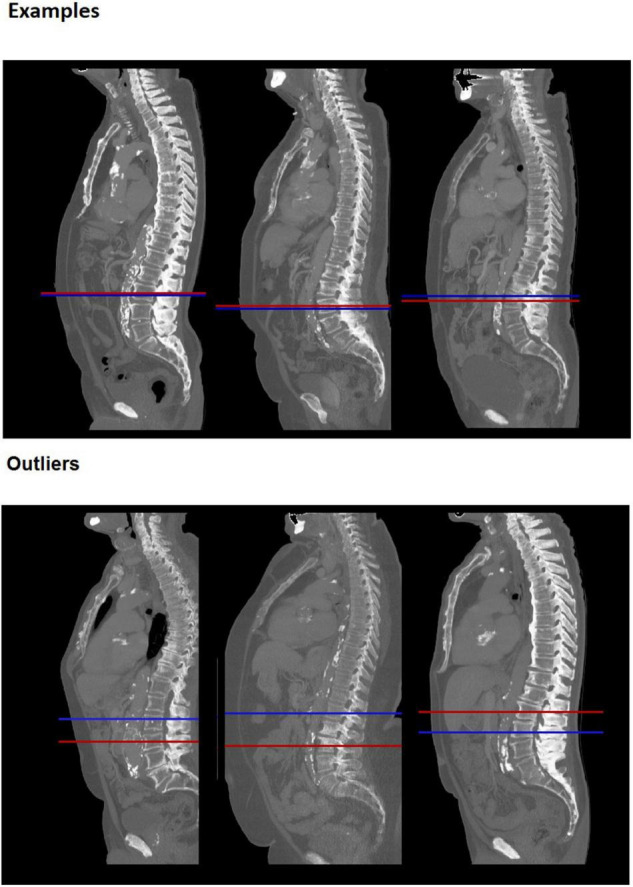
Examples of slice selection. Upper random examples and lower largest outliers, for 6 different patients. Red line: manually selected slice, Blue line: automatically selected slice. The bottom left and bottom middle example were incorrectly selected in the automatic analysis, which identified slices at L2 and L1 level, respectively. The bottom right example was incorrectly selected by the manual method and is correctly identified by the automatic analysis.

Median difference in slice selection was 7.5 mm [IQR: 3.0–16.5] and the bias was 3.4 mm with 95% limits of agreement of −24.5 to 31.4 mm. Visual inspection showed that 84% of the slices were on the same vertebra in both methods. The other 16% of the automatic selected slices were one slice higher or lower than the manual selected slice and one scan had an automatic selected slice two vertebra higher than the manual selected slice (Figure 2). Slice selection had an ICC of 0.86 [95% CI: 0.83–0.89] and a within-subject CV of 3.0% [95% CI: 2.8–3.3]. For slice selection male patients had a significantly lower within-subject CV than female patients, 2.7% [2.4–3.0] vs. 3.3% [2.9–3.7] (*p* = 0.01). There were no significant differences between BMI groups.

### Evaluation of Psoas Muscle Segmentation on the Manual Selected Slice

**FIGURE 3:**
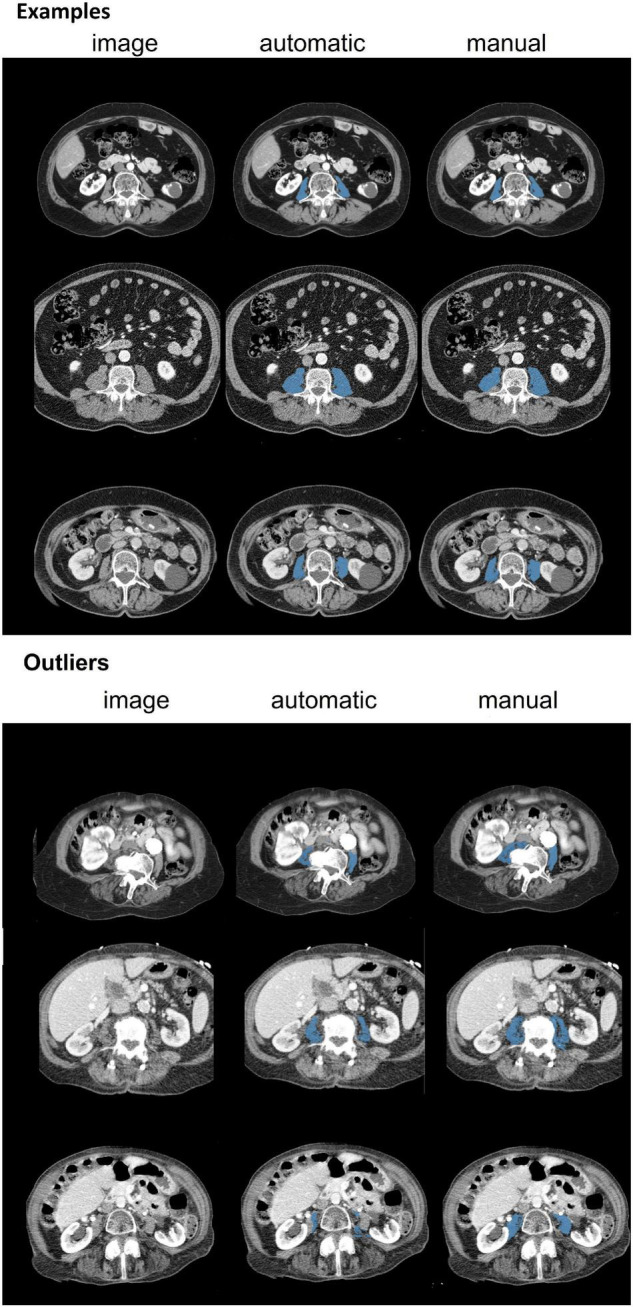
Examples of three randomly selected CT scans and three largest outliers for 6 different patients. Dice indices for these examples were 0.92 (top row), 0.90 (middle row) and 0.93 (bottom row). Dice indices for outliers were 0.74 (top row), 0.75 (middle row) and 0.36 (bottom row). The outlier shown in the top row contains an artifact, the middle row shows an overexposed vertebra and the bottom row shows an uncommon distribution of connective tissue, organs and muscle area and is incorrectly segmented by the automatic software.

Average psoas muscle area was 15.4 ± 4.6 cm^2^ for manual segmentation and 14.9 ± 4.6 cm^2^ for the automatic segmentation (Table 2). Median difference between methods was 0.69 cm^2^ [IQR:0.31–1.19], and Bland-Altman analysis (Figure 1) showed a bias of −0.55 cm^2^ with a 95% limit of agreement of −2.80 to 1.71 cm^2^ and an ICC of 0.96 [95% CI: 0.93–0.98]. Overall Dice index was 0.93 ± 0.04 and within-subject CV was 4.4% [95% CI: 4.0–4.8]. Examples of three randomly segmented CT scans and the three largest outliers, based on within-subject CV are shown in Figure 3. For male patients the Dice index was significantly higher 0.94 ± 0.03 vs. 0.92 ± 0.05 (*p* < 0.001) and the within-subject CV was significantly lower 3.7% [3.3–4.1] vs. 5.0% [4.4–5.6] (*p* < 0.001) than for female patients. Overweight and obese patients had significantly higher Dice index than patients in the normal/underweight group, 0.94 ± 0.03 and 0.94 ± 0.03 vs. 0.92 ± 0.06 (*p* < 0.001), respectively. The within-subject CV was significant lower in obese patients compared to the normal/underweight group, 3.6% [3.1–4.1] vs. 5.2% [4.3–6.0] (*p* = 0.008).

**TABLE 2:** Comparison between manual and automatic segmentation, slice selection and fully automatic assessment of psoas muscle area.

Full automatic	Manual PMA cm^2^	Automatic PMA cm^2^	Bias [95% Limit of agreement], cm^2^	ICC [95%CI]	CV [95%CI]	
Psoas muscle area	15.4 ± 4.6	14.7 ± 4.7	−0.69 [−6.60—5.23]	0.78 [0.74—0.82]	11.2% [10.2—12.2]	
**Gender**						
Male Female	18.6 ± 4.3 12.8 ± 2.9	18.1 ± 4.1 11.9 ± 3.0	−0.47 [−7.25–6.31] −0.86 [−5.93–4.21]	0.66 [0.59–0.73] 0.59 [0.49–0.68]	10.0% [8.6–11.4]^a^ 12.2% [10.8–13.6]^a^	
**BMI groups**						
Normal/underweight Overweight Obese	14.9 ± 4.2 15.6 ± 4.8 15.7 ± 4.7	14.6 ± 4.3 14.9 ± 4.7 14.7 ± 5.1	−0.39 [−6.14–5.36] −0.70 [−6.50–5.09] −1.05 [−7.32–5.23]	0.77 [0.70–0.82] 0.80 [0.74–0.85] 0.77 [0.68–0.84]	11.5% [9.6–13.4] 10.2% [9.0–11.4] 12.5% [10.6–14.4]	

**Slice selection only**	**Manual slice selection, mm**	**Automatic Slice selection, mm**	**Bias [95% Limit of agreement], cm^2^**	**ICC [95%CI]**	**CV [95%CI]**	

Slice selection L3	253 ± 28	256 ± 28	3.4 [−24.5–31.4]	0.86 [0.83–0.89]	3.0% [2.8–3.3]	
**Gender**						
Male Female	261 ± 26 246 ± 27	263 ± 28 251 ± 28	1.9 [−25.3–29.1] 4.7 [−23.7–33.1]	0.87 [0.83–0.89] 0.85 [0.80–0.89]	2.7% [2.4–3.0]^a^ 3.3% [2.9–3.7]^a^	
**BMI groups**						
Normal/underweight Overweight Obese	254 ± 27 253 ± 29 252 ± 26	253 ± 27 257 ± 29 259 ± 28	−0.9 [−29.0–27.2] 4.7 [−21.1–30.4] 7.0 [−21.9–35.9]	0.86 [0.82–0.89] 0.89 [0.84–0.92] 0.83 [0.71–0.89]	3.0% [2.6–3.4] 3.0% [2.5–3.2] 3.4% [3.0–3.7]	

**Segmentation only**	**Manual PMA, cm^2^**	**Automatic PMA, cm^2^**	**Bias [95% Limit of agreement], cm^2^**	**ICC [95%CI]**	**CV [95%CI]**	**Dice Index** **Mean ± SD**

Psoas muscle area	15.4 ± 4.6	14.9 ± 4.6	−0.55 [–2.80–1.71]	0.96 [0.93–0.98]	4.4% [4.0–4.8]	0.93 ± 0.04
**Gender**						
Male Female	18.6 ± 4.3 12.8 ± 2.9	18.1 ± 4.2 13.1 ± 3.0	−0.48 [−3.02–2.07] −0.61 [−2.59–1.38]	0.95 [0.92–0.96] 0.92 [0.82–0.96]	s3.7% [3.3–4.1]^a^ 5.0% [4.4–5.6]^a^	0.94 ± 0.03^a^ 0.92 ± 0.05^a^
**BMI groups**						
Normal/underweight Overweight Obese	14.9 ± 4.2 15.6 ± 4.8 15.7 ± 4.7	14.2 ± 4.2 15.1 ± 4.8 15.3 ± 4.8	−0.36 [−2.13–1.41] −0.49 [−2.85–1.88] −0.36 [−2.13–1.41]	0.94 [0.85–0.97] 0.96 [0.94–0.98] 0.98 [0.97–0.99]	5.2% [4.3–6.0] 4.3% [3.7–4.8] 3.6% [3.1–4.1] ^b^	0.92 ± 0.06 0.94 ± 0.03^b^ 0.94 ± 0.03^b^

*^a^CV significant different from other gender; ^b^significant different from normal/underweight BMI; ICC, intraclass correlation coefficient; CV, coefficient of variation; SD, standard deviation; C, confidence interval.*

## Discussion

The aim of the study was to compare fully automatic psoas muscle area measurements and manual psoas muscle area measurements on a CT scan at the level of L3 vertebra. The results showed that automatic slice selection identified slices on the same vertebra as the manual selection in 84% of the cases and otherwise was one vertebra higher of lower than manual selected slice. The Dice index for the psoas segmentation was high with a score of 0.93, which means that overlap between methods is on average 93%.

This is the first study to evaluate assessment of the psoas muscle at L3 level in CT scans with automatic deep learning-based software (12). Concurrent slice selection between the manual and automatic method was correct in a vast majority off the CT scans. The median difference was 7.5 mm which is well within the range of the L3 vertebra, with a height of approximately 30 mm (20). Given that there is no substantial change of psoas muscle area over the entire vertebra height at the L3 level (8), it can be expected that small variations in slice selection within the range of L3 vertebra do not lead to a systematic segmentation error (21, 22). Besides a good performance on slice selection, we also observed that the difference between the manual and automatic segmentation is comparable with the interobserver variability of the manual method performed by trained researchers (16, 23, 24). Indeed, multiple studies in TAVR and pancreatitis patients observed an interobserver ICC of approximately 0.97 between trained researchers, which is close to our observed ICC of 0.96 (16, 23, 24).

In-depth analysis showed differences in Dice index and within-subject CV between the manual and automatic method for sex and BMI. Male, overweight and obese patients had a higher Dice index and lower within-subject CV compared to the female and normal/underweight patients. These results could be explained by the larger psoas muscle area in the male and overweight patients. Incorrectly segmented voxels in one of the methods have relatively less impact in patients with a larger psoas muscle area. Furthermore, these patients have relatively less transition from the psoas to the quadratus lumborum and erector spinae, because this area is more prone to segmentation errors, less transition area leads to lower differences (8). Similar results have been found earlier in other studies, in which groups with larger psoas muscle area also showed a higher Dice index between methods (13). However, differences were only small and most likely not clinically significant.

The results of this study have important implications for research and clinical practice. Automatic software provides the possibility to process large sets of CT scans without high costs of time and expertise. Large scale studies can be performed on available routine clinical scans in various patient populations to search for imaging biomarkers that predict clinical outcomes such as total muscle area at the level of L3 vertebra, muscle quality which can be measured as muscle density, or intra- and intermuscular adipose tissue (25). Furthermore, segmentation of muscle mass and muscle quality can easily be performed over multiple slices. Future research can therefore focus on muscle volume instead of muscle area. Some studies with deep learning have already been performed correctly determining volume of iliopsoas (26). However, it is currently unknown if complete volume is also predictive of clinical outcomes. Therefore, our study first focused on complete manual slice selection and segmentation of psoas muscle area on single slice. For clinical practice the fully automatic method can offer tremendous opportunity, as it could be applied, with limited overhead, alongside any clinical protocol that includes abdominal CT. The automatic method makes it possible to use this data during daily clinical practice. The information about muscle mass can be used to identify frail, malnourished or sarcopenic patients at higher risk of adverse clinical outcomes, which is currently not used in clinical practice (1, 2). In TAVR patients, other surgical patients and community based older adults it is already shown that low muscle mass and low psoas muscle area are associated with negative clinical outcomes including length of stay, physical decline and mortality (3, 27, 28). Patients identified with low muscle mass can be selected to receive extra specialized care and preventive treatment including supervision from a dietician and physiotherapist to increase muscle mass, which is of interest for future research (29, 30).

This is the first study that applies deep learning-based software for automatic muscle assessment on the CT scan in a large cohort of elderly cardiac patients. In-depth analysis of the method comprised of slice selection and segmentation was performed providing a complete overview of the performance of the automatic muscle assessment. The study has some limitations. First, the comparison between automatic and manual segmentation was limited to psoas muscle area, while the software can also segment abdominal muscle area, long-spine muscle area, visceral fat area and subcutaneous fat area. However, the narrow field of view of the scans prohibited accurate analysis of these tissues in several patients. Second, the study is single center by design and includes only patients planned for TAVR, i.e., patients with severe aortic stenosis. However, the employed body composition method is trained using images from various patient populations and with various acquisition protocols (e.g., with and without contrast agents). It is likely that the performance of the method will generalize to other cohorts, because muscle composition is relatively similar among cohorts (31–33). However, this hypothesis should be confirmed in future studies, especially in groups with extreme or deviating muscle composition. Finally, manual segmentation and slice selection were used as reference standard. Although manual segmentation is generally an accepted method, which is validated and has good performances, manual slice selection and segmentation can contain human errors, as can be seen by the largest outliers in this study.

To conclude, fully automatic deep learning-based assessment of muscle area on a CT scan offers an accurate and reliable alternative for the currently used manual method. It is a tool that offers great opportunities for analysis in large scale studies and in clinical applications.

## Data Availability Statement

The dataset used in this study can be found in an online repository with digital object identifier: doi: 10.21942/uva.19403444.v1.

## Ethics Statement

The studies involving human participants were reviewed and approved by the METC AMC. The patients/participants provided their written informed consent to participate in this study.

## Author Contributions

DV, RD, and JS performed the first draft of the manuscript in close cooperation with all other authors. MV and YJ performed manual slice selection and segmentation. PM performed the automatic deep learning assessment of the psoas muscle area, both blinded for the contradicting method. DV performed the performance of all analysis. All authors did cooperate in the concept and design of the manuscript, interpretation of data and gave critical revision on the full manuscript.

## Conflict of Interest

PM was employed by Quantib-U. BD was Artificial Intelligence lead and cofounder of Quantib-U. II was a cofounder and Scientific lead at Quantib-U. The remaining authors declare that the research was conducted in the absence of any commercial or financial relationships that could be construed as a potential conflict of interest.

## Publisher’s Note

All claims expressed in this article are solely those of the authors and do not necessarily represent those of their affiliated organizations, or those of the publisher, the editors and the reviewers. Any product that may be evaluated in this article, or claim that may be made by its manufacturer, is not guaranteed or endorsed by the publisher.

## Footnotes

1
Research.quantib.com
